# Human Menstrual Blood-Derived Stromal Cells Promote Recovery of Premature Ovarian Insufficiency Via Regulating the ECM-Dependent FAK/AKT Signaling

**DOI:** 10.1007/s12015-018-9867-0

**Published:** 2018-12-17

**Authors:** Penghui Feng, Pingping Li, Jichun Tan

**Affiliations:** Department of Obstetrics and Gynecology-Reproductive Medical Center of Shengjing Hospital Affiliated to China Medical University, Shenyang, China

**Keywords:** Menstrual blood, Stromal cells, Premature ovarian insufficiency, Ovarian function

## Abstract

POI is characterized by “absent not abnormal” menstruation with hormonal disorders in woman younger than 40 years of age, and etiological and pathophysiological mechanisms underlying the POI development have not been clearly defined. Recently, due to advantages such as abundant sources and non-invasive methods of harvest, MenSCs have been emerging as a promising treatment strategy for the recovery of female reproductive damage. Here, we demonstrated that MenSCs graft in POI mice after CTX treatment could restore ovarian function by regulating normal follicle development and estrous cycle, reducing apoptosis in ovaries to maintain homeostasis of microenvironment and modulating serum sex hormones to a relatively normal status. Moreover, MenSCs participated in the activation of ovarian transcriptional expression in ECM-dependent FAK/AKT signaling pathway and thus restored ovarian function to a certain extent. MenSCs transplantation was proved to be an effective way to repair ovarian function with low immunogenicity, suggesting its great potential for POI treatment.

**Electronic supplementary material:**

The online version of this article (10.1007/s12015-018-9867-0) contains supplementary material, which is available to authorized users.

## Introduction

POI poses a considerable risk to female reproductive health [1, 2]. As a principal factor in female infertility, POI is diagnosed and characterized by oligomenorrhea or amenorrhea for at least four months prior to 40 years of age, and POI patients exhibit increased levels of FSH (> 25 U/L between two detections) [3, 4]. These changes may lead to increased risk of osteoporosis, emotional issues or cardiovascular diseases; ~1% of woman worldwide are affected by POI, and etiological and pathophysiological mechanisms underlying its development have yet not been fully known [5, 6]. Genetic [7, 8], immunological [9], and environmental factors [10, 11] have significant impact on POI development. In clinical applications, some interventions such as hormonal replacement therapy (using 17-β estradiol and dydrogesterone) [12, 13] and traditional Chinese medicine [14] are intended to relieve endocrine disturbance or restore ovarian function; however, the effectiveness of these measures is limited. Recently, the therapeutic potential of stem cells in POI treatment has gained extensive attention due to their multipotent differentiation ability and diverse sources such as chorionic plate, adipose tissue, umbilical cord, bone marrow, skin and even menstrual blood [15–19].

MenSCs isolated from menstrual blood exhibit typical MSCs-like characteristics including pluripotency, self-renewal potential and low immunogenicity [20, 21]. It has been reported that MenSCs were able to differentiate into osteocytes, chondrocytes, adipocytes, hepatocyte-like cells or oocyte-like cells in vitro [22–24]. Upon transplantation for damage repair and regeneration, MenSCs showed several advantages like non-invasive access and abundant sources over other MSCs. To date, several studies on MSCs have held the potential and promise to improve or restore ovarian function in animal models. In these studies, MSCs played a principal role in restoration of ovarian function in a paracrine manner by secreting some cytokines such as hepatocyte growth factor and epidermal growth factor [25–28]. In addition, MSCs were involved in regulation of immune system in POI [29]. However, the detailed mode of action of MenSCs on ovarian tissues has yet not been well understood until now.

Thus, in this study, we investigated the gene expression profile and the regulatory mechanisms occurring in ovarian tissues after MenSCs administration through the tail veins in CTX-induced POI mice and explored the reparative effect of MenSCs on ovarian function.

## Materials and Methods

### Isolation and Culture of MenSCs

For study purposes, samples were collected from six woman aged 25 ~ 35 years old with the informed consent approved by the Ethics Committee of Shengjing Hospital affiliated to China Medical University. MenSCs were then isolated and cultured accordingly as our previous study reported [30]. In brief, MenSCs were purified from menstrual blood by density gradient centrifugation with Ficoll-Paque solution (GE Healthcare Bio-sciences, Uppsala, Sweden). The enriched MenSCs were cultured in 25-cm^2^ culture flask with DMEM/F12 medium (Hyclone, Logan, UT, USA) supplemented with 10% FBS (Gibco, Waltham, MA, USA), 2 mM L-glutamine (Sigma-Aldrich), 1% non-essential amino acid (Sigma-Aldrich) and 1% penicillin-streptomycin (Sigma-Aldrich) in a humidified incubator under 37 °C with 5% CO_2_.

### Flow Cytometry for Phenotype Analysis

MenSCs of P2 were cultured in 100-mm culture dishes and harvested after rinsing with PBS. To analyze the surface markers, cells (about 1 × 10^6^ cells) were segregated into single cell suspension in 100 μl buffer with 2% bovine serum albumin and incubated with the following antibodies (detailed product information was included in the supplementary material in Table. S1): PE-conjugated antibodies against CD38, CD73, or CD90, and FITC-conjugated antibodies against CD34, CD44, CD45 or CD105 at 4 °C for 30 min. After rinsing with binding buffer, cells were centrifuged at 1200 r/min for 5 min and re-suspended with 0.5 ml binding buffer. The antibody-labeled cells were determined by FACSCalibur flow cytometry (BD Biosciences, San Jose, CA) and the data were analyzed with Flowjo 7.6.1 software.

### Multi-Lineage Differentiation Potential of MenSCs

In order to evaluate the differentiation potential of MenSCs, cells of P2 were induced into adipogenic, chondrogenic and osteogenic lineages.

For induction of adipogenic differentiation, MenSCs were seeded at six well plates at 2 × 10^4^ cells/cm^2^ in DMEM/F12 medium. When cells reached 100% confluence, OriCell™ (Cyagen, Suzhou, China) induction medium A (consisting of 0.1 μl/ml dexamethasone, 2 μl/ml insulin, 1 μl/ml IBMX and 1 μl/ml rosiglitazone) and medium B containing 2 μl/ml insulin were substituted in rotation as the manufacturer’s protocol. The whole process lasted for 21 days and cells were finally fixed with 4% paraformaldehyde followed by staining with Oil Red [31].

To evaluate chondrogenic differentiation, MenSCs were harvested and centrifuged to form a cellular pellet of 2.5 × 10^5^ cells, which was then kept in chondrogenic differentiation basal medium supplemented with 0.1 μl/ml dexamethasone, 3 μl/ml ascorbate, 10 μl/ml ITS + supplement, 1 μl/ml sodium pyruvate, 1 μl/ml proline and 10 μl/ml TGF-β3. Medium was replaced every three days and cells were maintained in humidified condition at 37 °C with 5% CO_2_. After 28 days, the induced chondrocyte spheroids were fixed with 4% paraformaldehyde and embedded with paraffin. Whereafter, paraffin sections underwent deparaffinization and staining with Alcian Blue dye for 30 min [32]. Sections were eventually analyzed under microscope.

To assess osteogenic induction, cells were plated and cultured at 2 × 10^4^ cells/cm^2^ in six well plates until they reached 60% confluence with 0.1% gelatin solution precoated for 30 min. Then, cells were induced by osteogenic differentiation medium with 2 μl/ml ascorbate, 10 μl/ml β glycerophosphate sodium and 0.1 μl/ml dexamethasone. Medium was changed every three days. About 28 days later, cells were detected by Alizarin red staining after fixation with 4% paraformaldehyde [33].

### Cell Proliferation Assay

MenSCs of P2 and P10 were seeded in 96 well plates (Corning, New York, USA). Cells were cultured at approximately 2 × 10^3^ cells/well in DMEM/F12 medium supplemented with 10% FBS, 2 mM L-glutamine, 1% non-essential amino acid and 1% penicillin-streptomycin and medium was refreshed every three days. On each day of the following 10 days, the original medium was replaced with 100 μl DMEM/F12 medium and then 10 μl CCK-8 solution (Beyotime, Beijing, China) was added for OD value, which was measured after incubation for 2 h by reading absorbance at 450 nm by microplate reader (Sunrise RC, Tecan Group, Maennedorf, Switzerland).

### Colony-Forming Assay

MenSCs of P2 and P10 were seeded onto 100-mm cell culture dishes respectively at the density of 150 cells/cm^2^. Cells were cultured in DMEM/F12 medium supplemented with 10% FBS, 2 mM L-glutamine, 1% non-essential amino acid and 1% penicillin-streptomycin and medium was refreshed every three days. After 10 days, the cells were washed with precooled PBS and then fixed in 4% paraformaldehyde. Results were observed with Wright Giemsa staining under microscope. Colony-forming units were defined and counted according to colonies with no less than 50 cells.

### Animal Model of POI and MenSCs Xenograft

Female C57BL/6 mice (*n* = 150) of six weeks were purchased from Beijing Vital River Laboratory Animal Technology Co., Ltd. (Beijing, China) and experiments performed were approved in accordance with the Guide for the Care and Use of Laboratory Animals and Animal Use and Care Committee of China Medical University. Mice were maintained for one week to aid acclimatization in specific pathogen-free condition with temperature controlled and free access to food and water under standard dark-light cycle. In order to build POI models, mice underwent intraperitoneal injection of 70 mg/kg CTX (Sigma-Aldrich) at age of seven weeks [34]. Mice were randomly divided into three groups: group NO (normal wild-type mice without CTX treatment, *n* = 50), group MO (mice were injected with CTX as POI model, n = 50) and group ME (mice were grafted with 500 μl cells suspension containing 1 × 10^6^ MenSCs through tail vein injection in seven days after CTX treatment, n = 50) [26, 35].

During the process, vaginal smears were carried out daily for detecting the change of estrous cycle under microscope. According to the cytological components of keratinized epithelium, lymphocytes and nucleated epithelial cells, estrous cycles of mice were clarified into four stages. On the tenth day after cells transplantation, 38 mice of each group were sacrificed to collect ovaries, serum samples and uteri for experimental detection. Six mice of each group were arranged for vaginal smears observation. At the same time, the rest of the female mice from each group were mated with proven fertile male mice (1:1) for two weeks and pregnancy was confirmed after the presence of vaginal in the cage. After that, female mice were fed separately until their delivery. Live births of their offspring were recorded in this fertility test.

### Vaginal Cytology

Estrous cycles of female mice were tracked to exclude from the study at the beginning those mice whose cycles were abnormal (average cycle length was out of 4 to 6 days or cycles with discontinuous stages). In brief, vaginal smears were obtained daily, conducted with vaginal flushing fluid of 10 μl PBS. Samples were then stained with Wright-Giemsa’s stain (Tbdscience, Tianjin, China) according to standard protocols and scored under light microscope. Smears which contained mainly nucleated cells were defined as proestrus, and smears which were primarily composed of non-keratinized squamous cells without or with little leukocytes were classified as estrus. In addition, samples predominantly consisted of keratinized squamous cells and abundant leukocytes were regarded as metaestrus. In terms of diestrus, smears were almost leukocytes in the whole scene [36, 37].

### Morphological Observation and Ovarian Follicle Count

Ovaries collected from mice after CTX administration and MenSCs graft were washed by precooled PBS for three times, followed by fixation in 4% paraformaldehyde overnight. Then ovarian tissues were eventually sectioned at 4 μm thick and mounted on glass slides following dehydration and paraffin-embedding. After that, routine H&E staining was performed for histopathological analysis under light microscope. To estimate follicles in each ovary, six representative ovarian sections were selected and only follicle containing an oocyte was counted to avoid counting repeatedly. Follicles were defined according to the presence or distribution of granulosa cells, follicular antrum or cumulus cells as follows [38, 39]. Primordial follicles were identified when oocyte was surrounded by a continuous or non-continuous single layer granulosa cells, but it was cuboidal shape of granulosa cells that was shown in primary follicles. Secondary follicles were classified as there were no less than two layers of cuboidal cells in follicles without antral space, whereas antral follicles possessed antral space formed by follicular fluid. Preovulatory follicles generally had a rim of cumulus cells surrounding oocyte.

### Enzyme Linked Immunosorbent Assay

Mice serum was isolated from blood samples by eyeball extracting, which coagulated at room temperature for one hour and were centrifuged at 1500 r/min for ten min. Subsequently, mice Elisa panel kits were performed for detection of E_2_, FSH and AMH (Cusabio, Wuhan, China). On the basis of the kits’ instructions, mice E_2_, FSH and AMH standards were diluted as recommended concentration ranges of 25 pg/ml ~ 1000 pg/ml, 4 mIU/ml ~ 140 mIU/ml and 0.4 ng/ml ~ 14 ng/ml, respectively. This assay employed the competitive inhibition enzyme immunoassay technique, and the competitive inhibition reactions were launched between HRP labeled hormones and unlabeled hormones with the antibody. The substrate solution of tetramethyl benzidine was added and the final results were detected at 450 nm wave length and measured using the microplate reader.

### Apoptosis Assay

Ovarian sections were disposed with Hoechst kit (Wanleibio, Beijing, China), staining for ten min in the dark. Fragmented or condensed nuclei of apoptotic cells were observed using fluorescence microscope. TUNEL assay was performed using TUNEL FITC Apoptosis Detection Kit (Vazyme, Nanjing, China). Tissue permeability was enhanced with 20 μg/ml proteinase K. Samples were then incubated with terminal deoxynucleotidyl transferase and FITC-12-dUTP Labeling Mix buffer. Tissue sections were observed for positive cells by using fluorescence microscope.

### Quantitative Real-Time PCR Analysis

Expression levels of mRNA was evaluated by q-RT PCR and total RNA was extracted from ovarian tissues by Trizol Reagent (Takara, Japan) according to the manufacturer’s direction. Reverse transcription was performed to generate cDNA strand preconditioned with gDNA Eraser (PrimeScript™ RT reagent Kit with gDNA Eraser (Perfect Real Time), Takara). Real-time PCR was conducted by using SYBR® Premix Ex Taq™ (Takara, Japan). PCR primer sequences were designed by primer premier 6.0 software (http://www.premierbiosoft.com/products/products.html) as listed in Table 1. In addition, cycling conditions for the PCR machine (Applied Biosystems 7500 Real Time PCR System) were set as follows: 95 °C for 5 s and 60 °C for 34 s for 40 cycles in a 20 μl reaction volume. Gene expression levels were assessed by using the delta-delta CT method and were standardized to that of GAPDH RNA amplification levels.

**Table 1:** PCR primer list

Gene	Sequence (5′ - 3′)
AMH	F: GTTGCTAGTCCTACATCTGGCTGAA
	R: CAGGTGGAGGCTCTTGGAACTTC
DDX4	F: GCTGCCGTGGAGGATTTGGTCTA
	R: GCCTGAATCACTTGCTGCTGGTT
VEGFA	F: TTCGGGAACCAGACCTCTCA
	R: GACCCAAAGTGCTCCTCGAA
COL6A5	F: CAGTGGGGACCACACAGAAT
	R: CCCCAACATTAGATGCCCCA
COL9A2	F: CAGCCTATACCTCTGCTCGC
	R: CCCCTGGATCTTGGGTTTCC
GAPDH	F: GTGAAGGTCGGTGTGAACGGATT
	R: GGTCTCGCTCCTGGAAGATGGT

### Western Blotting Analysis

Detailed procedure for western blotting analysis was described in supplementary file (File. S1). Information of corresponding antibodies was included in the supplementary material in Table. S1.

### Immunohistochemistry

IHC staining was performed with primary antibodies against COL6A5 and COL9A2 following standard protocols that 4 μm paraffin sections of samples mounted on glass slides were successively deparaffinized, dehydrated and immersed in 3% H_2_O_2_ to block endogenous peroxidase activity. After rinsing with PBS, samples underwent heat-induced antigen retrieval by citric acid buffer, followed by incubation with primary antibodies overnight at 4 °C after blocking by goat serum. For further operation, SPlink Detection Kits (Biotin-Streptavidin HRP Detection Systems) (Zsbio, China) and DAB kits (MXB Biotechnologies, China) were used to detect to strength of positivity which was semi-qualified by proportion and intensity of brown cells in specimen.

### RNA Sequencing and Bioinformatic Analysis

RNA of ovarian tissues from Mo group and Me group was purified by Trizol reagent (Takara, Japan), and then mRNA was reversely transcribed into cDNA fragments after being quantified by the Nanodrop spectrophotometer (Thermo Scientific, USA). Library construction was performed by Illumina’s TruSeq RNA Library Prep Kit following standard protocols (Illumina, San Diego, CA) that mRNA was enriched by oligo (dT) magnetic beads, mRNA was fragmented with fragmentation buffer added, double cDNA was then synthesized using random hexamer-primers, dNTPs, buffer plus DNA polymerase I on the template of fragmented mRNA, and purified double cDNA strands were finally performed after end reparation, single nucleotide A (adenine) addition and sequencing adaptors ligation. Sequentially, mRNA-seq library was then sequenced on Illumina HiSeq 2500. Standard bioinformatics analysis was performed to identify DEGs which were selected when meeting the requirements of *P* < 0.05 and fold change >2 or < 0.5 between groups.

### Statistical Analysis

Data from at least three independent experiments were represented as mean ± SEM. One-way analysis of variance, Kruskal Wallis test and unpaired *t* test were applied to evaluate significance via IBM SPSS Statistics 22 software. ^*^P < 0.05 was considered statistically significant or ^**^P < 0.01 was considered to represent extremely significant differences for all statistical calculations.

## Results

### Isolation and Characterization of MenSCs

Microscopic observation demonstrated that MenSCs isolated from menstrual blood possessed typical spindle-like shape (supplementary file, Fig. S1A). To analyze the expression of surface markers on MenSCs, FCM was conducted as shown in Fig. S1B and MenSCs presented a low expression of hematopoietic cells antigens of CD45, CD38 and CD34 but a high expression of classical MSCs surface markers as CD105, CD90, CD73 and CD44 on the contrary. To evaluate multi-lineage differentiation potential of MenSCs, we cultured MenSCs of P2 in adipogenic, chondrogenic and osteogenic induction medium. Notably, lipid droplet formation was observed after staining with Oil Red O (Fig. S1C). Cells could be successfully induced into chondrocyte spheroids, stained with Alcian Blue dye (Fig. S1D). It was well to reveal that MenSCs were able to be induced into osteoblasts which could be visualized via Alizarin red staining (Fig. S1E).

For further characteristics identification, we compared proliferation ability and CFU formation ability of MenSCs of P2 and P10, we found that cells’ proliferation ability was not affected so apparently and cells entered into a quick proliferation stage at about day two and experienced a stationary period at about day eight as displayed in Fig. S1F. Similarly, Fig. S1G showed a typical colony under microscope, and results in Fig. S1H indicated that cell generation of P10 had not cause significant difference on self-renewable ability of MenSCs (*P* = 0.222). In summary, all these results demonstrated MenSCs exhibited typical characteristics as MSCs in terms of phenotypic correlation.

### MenSCs Transplantation Promoted Recovery of CTX-Induced POI

**Fig. 1:**
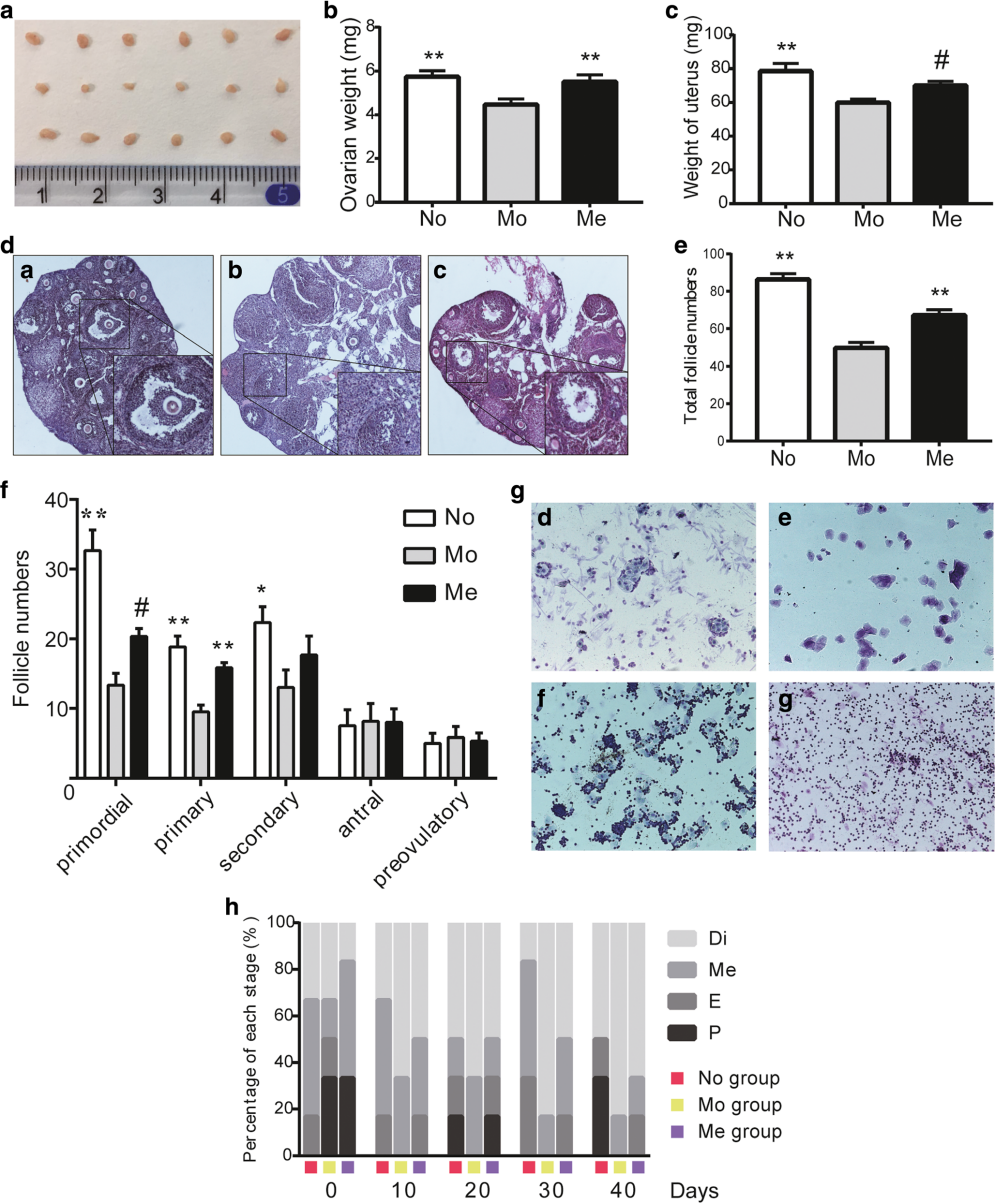
Direct effects of CTX injection and MenSCs transplantation on ovarian changes and estrous cycles. **a** The macroscopical changes of ovaries in No, Mo and Me groups from top to bottom after ten days since MenSCs graft. **b** Changes of ovarian weight among the three groups, *n* = 20 (data were represented as mean ± SEM, ^**^P < 0.01). **c** Changes of uterine weight among the three groups, n = 20 (data were represented as mean ± SEM, ^#^*P =* 0.051, ^**^P < 0.01). **c** Representative images of ovarian histological sections in No group (*a*), Mo group (*b*) and Me group (*c*). Original magnification, ×100. **d** The statistics of total follicles in each group, *n* = 6 (data were represented as mean ± SEM, ^**^P < 0.01). **e** Changes in numbers of primordial, primary, secondary, antral and preovulatory follicles in each group, n = 6 (data were represented as mean ± SEM, ^#^*P =* 0.056, ^*^P < 0.05, ^**^P < 0.01). **f** Representative images of estrous cycles in female mice including proestrus (*d*), estrus (*e*), metaestrus (*f*) and diestrus (*g*). Original magnification, ×100. **g** Variation trends of estrous cycles recorded every ten days until the fortieth day since MenSCs graft in No, Mo and Me groups, respectively, n = 6

POI mice induced by CTX were sacrificed on the tenth day to collect such target tissue or organ samples as ovaries, uteri and blood samples. In order to evaluate the effect of MenSCs on ovarian function, we first compared the ovarian size and weight changes among these three groups (No, Mo and Me groups). Consequently, we observed ovarian weight increased after cells therapy compared with that of CTX-induced Mo group (*P* = 0.018) as indicated in Fig. 1a, b. However, changes were not so statistically significant when it came to weight of uteri between group Mo and group Me (*P* = 0.051) (Fig. 1c). Furthermore, pathological analysis revealed that cytotoxic chemotherapeutic induction by CTX could lead to disturbance in follicle development in both quantity and quality while MenSCs transplantation could restore the follicle injury partially in follicle total numbers (49.83 ± 2.97 versus 67.17 ± 2.94, *P* = 0.002) (Fig. 1d, e). In detail, the quantitative statistics of various follicle numbers showed that primary follicles (*P* = 0.0029) were obviously increased in Me group in comparison with that of Me group whereas this difference was not so significant in primordial (*P* = 0.056). Besides, there was no difference between Mo and Me group in secondary follicles, antral and preovulatory follicles (*P* = 0.300*,* 0.998 and 0.955, respectively) (Fig. 1f).

To assess the estrous cycles, vaginal smears were operated and recorded every ten days until the fortieth day. Figures of proestrus, estrus, metaestrus and diestrus (Fig. 1g *d*, *e*, f and *g*, respectively) were shown as typical represents mainly identified by the presence and proportion of nucleated cells, keratinized or non-keratinized squamous cells and leukocytes. Moreover, estrous cycles of mice in Mo group were prone to be arrested on diestrus when compared with No group, but this situation could be relieved in part after treatment of MenSCs (Fig. 1h).

**Fig. 2:**
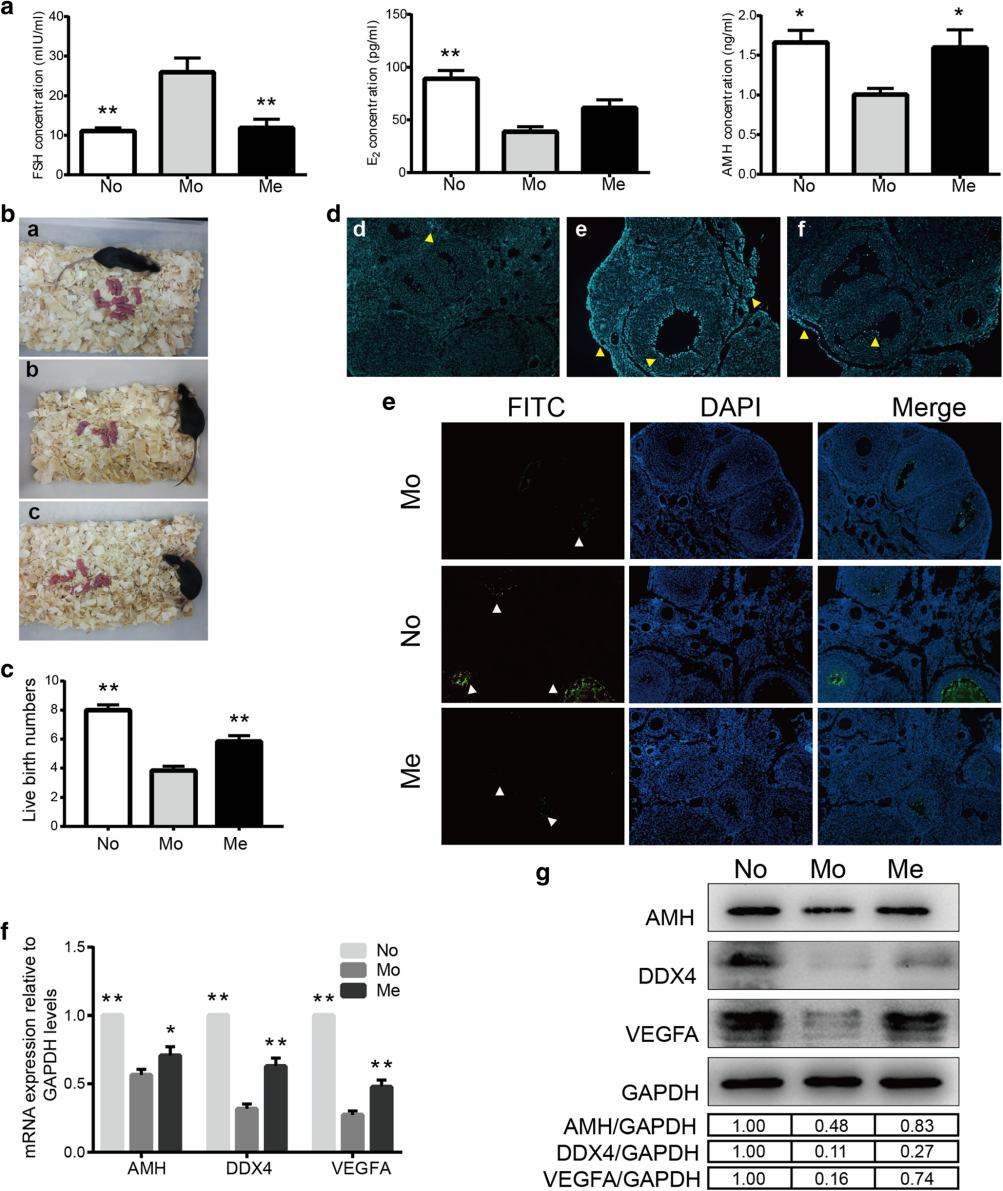
Physiological changes to evaluate ovarian status. **a** Serum hormone levels assessed by ELISA kits in each group for measuring FSH (left), E2 (middle) and AMH (right), n = 6 (data were represented as mean ± SEM, ^*^P < 0.05, ^**^P < 0.01). **b** Representative images of parturition in each group after mating with male mice. **c** Records of live births after CTX injection followed by MenSCs transplantation, n = 6 (data were represented as mean ± SEM, ^**^P < 0.01). **d** Apoptosis evaluation using Hoechst kit. Yellow arrows pointed to apoptotic cells with fragmented or condensed nuclei of apoptotic cells. Original magnification, ×100. **e** Apoptosis evaluation performed by TUNEL assay. White arrows pointed to FITC-labeled apoptotic cells. Original magnification, ×100. **f** Relative mRNA expression by q-RT PCR analysis for AMH, DDX4 and VEGFA controlled to GAPDH with fold change measured by 2^-ΔΔCT^, *n* = 6. **g** Relative expression at protein levels of AMH, DDX4 and VEGFA (upper panel) and quantitative analysis (lower panel)

Serum sample was obtained from each mouse when time was due on the tenth day after MenSCs transplantation. The therapeutic effect of MenSCs was evaluated by examining the serum hormone levels across the three groups (No, Mo and Me groups). Results indicated that serum FSH levels of Mo group were significantly increased compared to either No group (*P* = 0.001) or Me group (*P* = 0.002). In other words, MenSCs were capable to regulate FSH levels down to normal conditions. Additionally, serum E_2_ levels appeared to be reduced after CTX injection in comparison with No group (*P* = 0.001) and increased after MenSCs injection even though the difference was not significant (*P* = 0.198). Meanwhile, the changes of AMH levels demonstrated similar trend as E_2_ levels among these three groups, that AMH levels were decreased in Mo group (*P* = 0.023) but regained in Me group (*P* = 0.039) (Fig. 2a). Briefly speaking, ELISA results indicated a positive effect of MenSCs transplantation when the homeostasis of hormones in vivo was broken by CTX. This detrimental effect could be eliminated to a certain extent via the regulation of MenSCs.

In analysis of the final live births of female mice after mating with male mice since the tenth day after cells injection, live births of mice were recorded. Results were displayed of No, Mo and Me groups (Fig. 2b *a*, *b* and *c*, respectively). Data statistics revealed less live births after CTX treatment than No group (*P* < 0.001) and Me group with statistical significance (*P* = 0.001) (Fig. 2c).

For further comparison of ovarian function among these groups, Hoechst assay and TUNEL assay were carried out to determine apoptotic cells in ovarian tissues. Results brought up such a hint that there were fairly more apoptotic cells existing in Mo group which were mainly composed of granulosa cells than No group and MenSCs could dramatically reduce the occurrence of apoptosis (Fig. 2d, e).

Besides, qRT-PCR and western blotting were performed to investigate some functional molecular indicators including AMH, DDX4 and VEGFA. In terms of transcriptional expression, data manifested that CTX could inhibit the expression of these three molecules when compared with No group. Conversely, MenSCs graft could increase the expression of AMH, DDX4 and VEGFA as shown in Fig. 2f (*P* = 0.040, < 0.001 and = 0.001, respectively). Besides, these changes had the same tendency at protein level that AMH, DDX4, and VEGF were consistent with their mRNA levels (Fig. 2g).

All these results indicated that MenSCs transplantation could save ovarian function certainly from serious CTX damage. MenSCs came into effect from several aspects, namely, regulation of body hormone homeostasis, promotion of normal follicle development, restoration of rhythmicity of estrous cycles, relief in apoptosis of ovarian cells and modulation of related functional molecules’ expression as well. Taking the whole factors into consideration, it was suggested that MenSCs possessed great therapeutic potential for improvement in ovarian function and attenuation actions with chemotherapeutics.

### Transcriptomics Analysis for Differential Expression of mRNA after MenSCs Transplantation

**Fig. 3:**
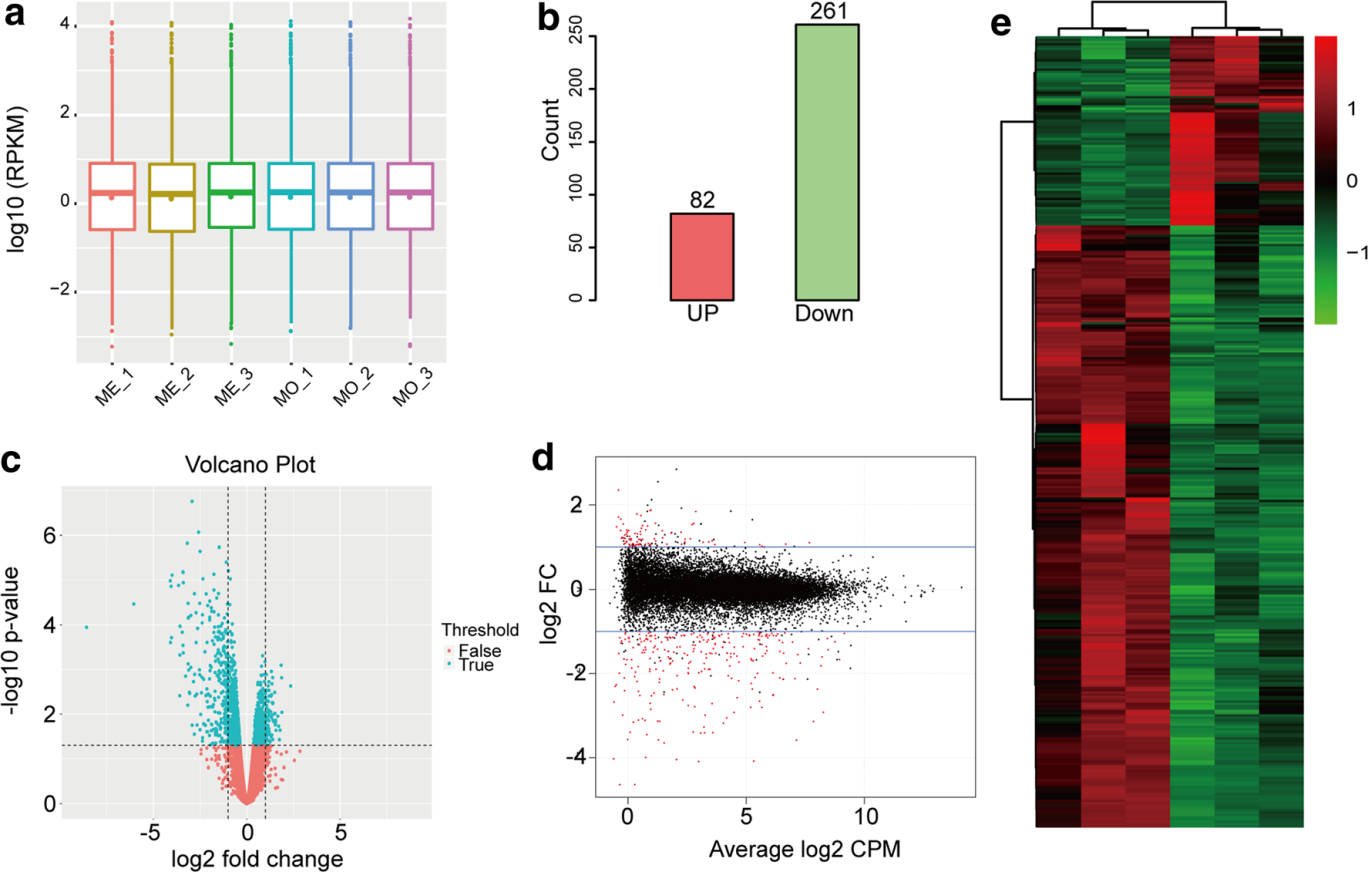
DEGs detection with mRNA sequencing analysis. **a** Boxplot of RPKM value of each sample in Mo and Me groups, showing similar expression levels of genes. **b** Statistics of DEGs basing on *P* < 0.05 and fold change >2 or < 0.5 when comparing Mo group with Me group. **c** Statistical analysis result plot for DEGs as volcano plot. **d** Statistical analysis result plot for DEGs as M-A plot. **e** Hierarchical clustering group heatmap of DEGs screened on the basis of *P* < 0.05 and fold change >2 or < 0.5. Red revealed genes that were relatively up-regulated, while green indicated down-regulated genes and black meant not statistically significant

**Fig. 4:**
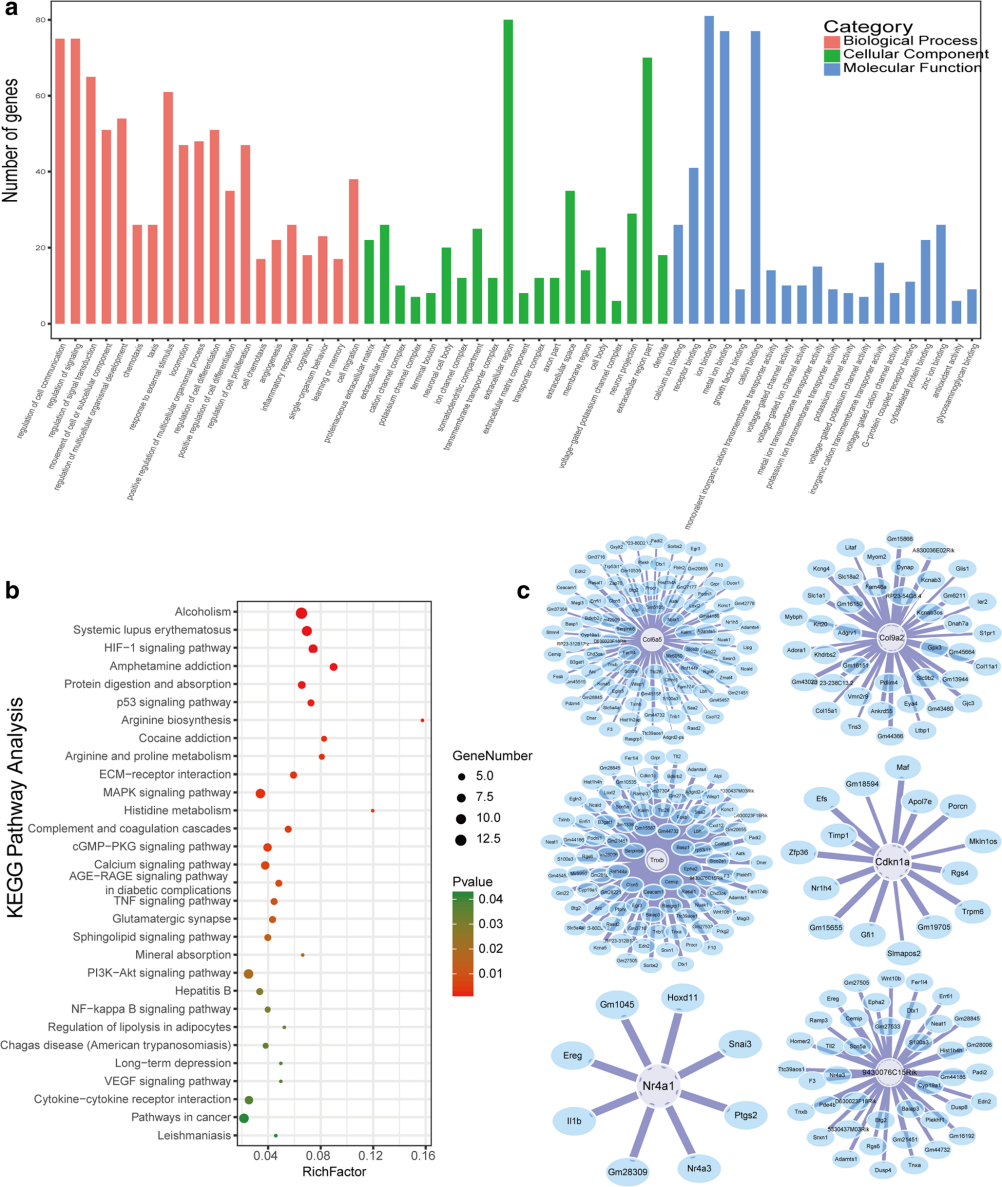
Go enrichment and signaling regulatory modules analysis of DEGs. **a** Go enrichment analysis including biological process, cellular component and molecular function. **b** KEGG pathway analysis of DEGs between Mo group and Me group. **c** Gene-co-expression network to analyze interaction of DEGs with *r* > 0.95 or < −0.95. The core genes were selected from DEGs which were involved in such pathways we concerned as ECM-receptor, focal adhesion and PI3K-AKT signaling pathways

To determine the effect of MenSCs on ovarian function at transcriptional levels under conditions of reproductive toxicity of CTX in POI mice, we performed mRNA sequencing. Data analysis revealed that Reads per Kilobase per Million Reads of total genes were similar in different samples of the two groups as the boxplot shown in Fig. 3a. Among these samples of the two groups, high-throughput mRNA sequencing (Fold change >2 or < 0.5, *p* < 0.05) yielded a total of 343 DEGs with 82 genes up-regulated and 261 genes down-regulated in Me group compared with Mo group. The DEGs from the two sets of samples data were shown as volcano plot and MA plot in Fig. 3c, d, respectively, on the basis of *P* value, fold change and counts per million reads. And the cluster analysis of heatmap of DEGs was shown in Fig. e. So as to investigate the mainly biological functions involved of DEGs in ovarian tissue, an integrated analysis of sequencing data was introduced via GO annotation analysis (http://amigo.geneontology.org/amigo) and KEGG enrichment platforms (http://kobas.cbi.pku.edu.cn/anno_iden.php). GO analysis results of DEGs were classified into three functional groups, including biological process, cellular component, and molecular function. In the biological process group, the DEGs were primarily enriched in, for instances, regulation of cell communication, regulation of signaling, chemotaxis, positive regulation of multicellular organismal process and angiogenesis as well. On the part of cellular component, the DEGs were mainly enriched in extracellular matrix, extracellular region, extracellular space, neuron projection and extracellular part. As for molecular function, the DEGs were principally enriched in receptor binding, iron binding, cation binding, cytoskeleton protein binding and zinc iron binding (Fig. 4a). Furthermore, KEGG analysis results indicated that these DEGs might take part in the pathways of HIF-1 signaling pathway, ECM-receptor interaction, P53 signaling pathway, PI3K-AKT signaling pathway, Focal adhesion, MAPK signaling pathway, Hippo signaling pathway, VEGF signaling pathway and so on. Results were shown in Fig. 4b.

For further study on the relationship among these DEGs identified from ovarian tissue in Mo and Me groups, we constructed gene-co-expression network to analyze interaction of DEGs via calculating the correlation coefficient of *r* value after the gene expression matrix of DEGs was established. After that, genes those with *r* > 0.95 or < −0.95 were screened to be used to predict putative regulatory signals in this RNA sequencing. We subsequently selected genes that we were concerned about and that were enriched in ECM-receptor interaction, Focal adhesion and PI3K-AKT signaling pathway. Data were finally imported into Cytoscape software (http://www.cytoscape.org/) to calculate the topological characteristics of the gene network (Fig. 4c).

### ECM-Dependent FAK/AKT Signaling Was Involved in Ovarian Function Regulation by MenSCs

**Fig. 5:**
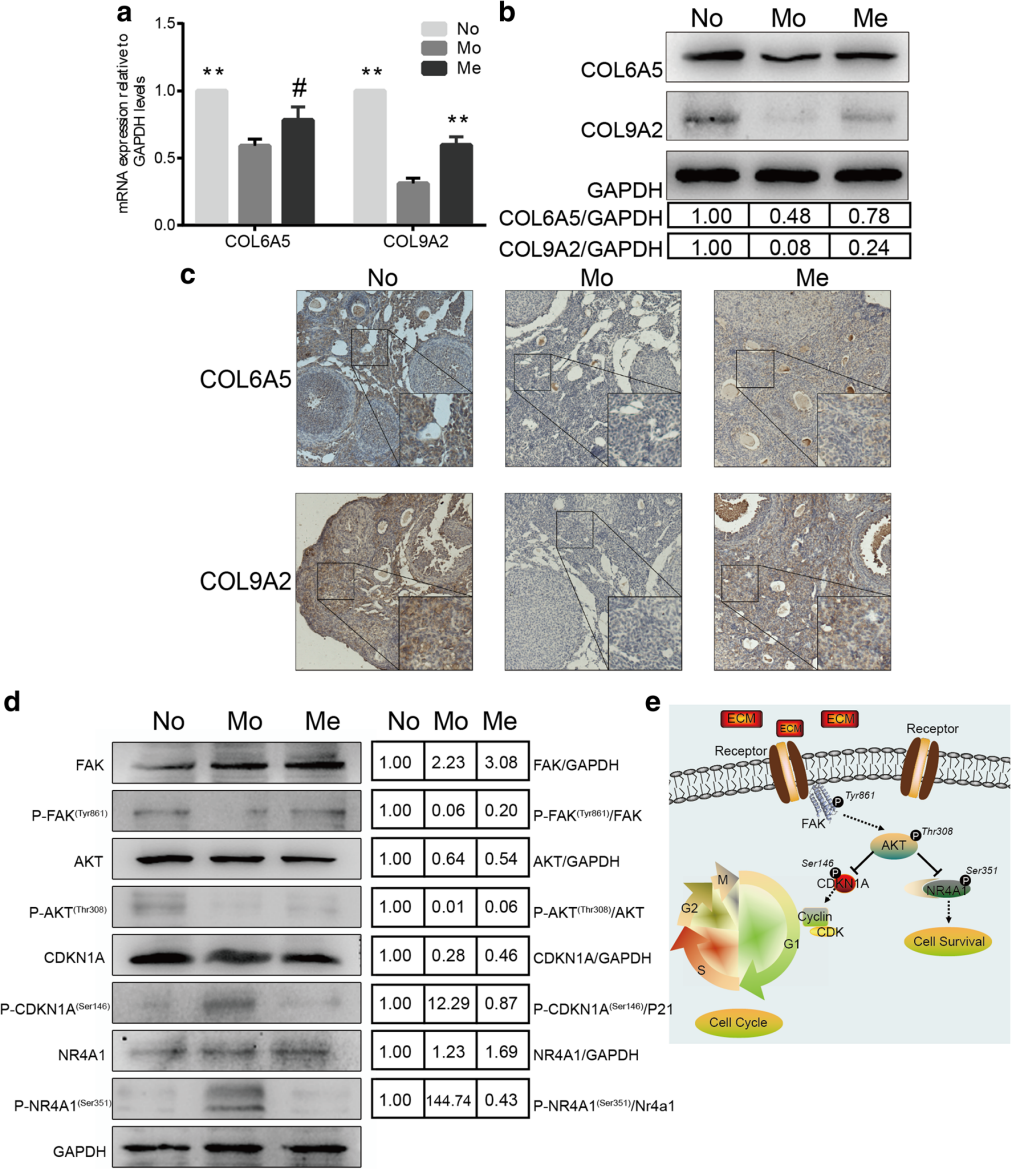
Signaling pathway validation. **a** Relative mRNA expression by q-RT PCR analysis of extracellular molecules of COL6A5 and COL9A2 in No, Mo and Me groups, n = 6 (data were represented as mean ± SEM, ^#^*P =* 0.052, ^**^P < 0.01). **b** Western blotting analysis for COL6A5 and COL9A2 expression (upper panel) and quantitative analysis (lower panel). **c** IHC analysis for detecting the expression of COL6A5 and COL9A2. **d** Western blotting analysis for FAK, AKT, CDKN1A, NR4A1 and their phosphorylated proteins (left panel) and quantitative gray level analysis (right panel)

To explore and confirm the potential molecular mechanism that participated in repair of ovarian function, we further verified related molecules expression based on the analysis of mRNA sequencing results and bioinformatic analysis. Firstly, we compared the mRNA expression of extracellular matrix molecules, COL6A5 and COL9A among the three groups (No, Mo and Me group). As shown in Fig. 5a, mRNA expression was elevated when comparing to Mo group in COL9A2 (*P* < 0.001) significantly after cells graft while COL6A5 appeared similar trend but not statistically different (*P* = 0.052). Interestingly, both of the two molecules, COL6A5 and COL9A2, were up-regulated in Me group and down-regulated in Mo group at protein levels; that was to say, CTX could reduce the expression of COL6A5 and COL9A2 while MenSCs could eliminate this negative effect partly as western blot results demonstrated (Fig. 5b) and IHC indicated (Fig. 5c). When further detecting downstream molecules in FAK-AKT signaling pathway in ovarian tissue after CTX injection and MenSCs transplantation, we found that phospho-FAK (Try861) and phospho-AKT (Thr308) proteins were increased after MenSCs treatment compared with Mo group; at the same time, the expression of phospho-CKDN1A (Ser146) and phospho-NR4A1 (Ser351) which were finally involved in cell cycles or survive were obviously decreased as shown in Fig. 5d. In total, all these results indicated that ECM-dependent FAK-AKT signals went through some changes that were probably involved in regulation of ovarian function of MenSCs as shown in Fig. 5e.

## Discussion

To date, effective ways to treat infertility due to POI in woman are still not available, even though some treatments are given clinically [40, 41]. Fortunately, with the development of regenerative medicine, excellent therapeutic prospects of MSCs have been predicted to repair tissues [42]. MenSCs, as a type of MSCs, exhibit all novel characteristics of MSCs and emerge as a suitable source of MSCs for tissue repair. By contrast to other MSCs like bone marrow-derived mesenchymal stem cells or adipose tissue-derived stromal cells, the process to harvest MenSCs is definitely non-invasive. Based on their low immunogenicity and non-invasiveness, MenSCs have been safely applied in such diseases as lung acute injury and uterine adhesion [30, 43, 44].

As previously reported [45], chemotherapeutic drugs like CTX could cause serious and prolonged damage to ovarian function in either human beings or animals. Additionally, CTX could induce apoptosis of granulosa cells, disturbing the homeostasis of ovarian microenvironment, and thus cause a detrimental impact on follicle development [46]. In the present study, we explored the effects of MenSCs on POI mice. Several studies have clearly indicated that many kinds of MSCs could promote follicle development in direct or indirect ways like paracrine, exosome or immune modulation [29, 47]. In accord with these findings, the observed outcomes in our study revealed that MenSCs graft could remarkably promote the regulation of normal follicle development and estrous cycle and modulate sex hormones including FSH, E_2_ and AMH. The levels of these hormones are novel parameters to represent ovarian reserve and menopausal state [48]. In POI patients or animals, ovarian atrophy, as observed in this study, could cause the decline in ovarian function and result in the relatively low levels of E_2_ and AMH, measured by ELISA assay. As a feedback mechanism, the decreased E_2_ would stimulate the pituitary gland to secrete higher levels of FSH. Furthermore, our findings demonstrated that MenSCs transplantation could reduce cellular apoptosis in ovarian tissues and increase molecular marker expression in ovaries including AMH, DDX4 and VEGFA in q-RT PCR and western blotting assays. Among them, AMH as a member of TGF-β superfamily proteins is synthesized and secreted by granulosa cells mainly in primary and antral follicles [49]. AMH is considered as a reliable marker of ovarian storage [50] as AMH is independent of hypothalamic-pituitary-gonadal axis function. And usually, AMH level is strongly correlated with the size of growing follicle [51]. As for DDX4, it presents in gonadal germ cells in both fetal and adult. DDX4 is abundant mostly in mature, as known, oocytes and spermatocytes and play a vital role in germ line cells development in ovary [52]. VEGFA is essential for both physiological and pathological angiogenesis. Also, VEGFA is necessary for vascular pattern development and endothelial cells differentiation [53]. In other words, the expression of VEGFA has a great impact on angiogenesis and blood supply and the latter offers ovaries an important guarantee for nutrients support and hormones transport. In general, these three markers could be regarded as “sentinels” for ovarian function in the aspects of follicle development, germ cells status and ovarian blood supply.

To further investigate the potentially transcriptional regulatory mechanisms occurring in ovaries after MenSCs administration, we performed mRNA sequencing and the findings demonstrated that many biological functions and signaling pathways were involved in ovarian function modulation as ECM-receptor interaction, focal adhesion, and PI3K-AKT signaling pathway. Then, we detected the expression of candidate molecules including COL6A5, COL9A2, and downstream molecules that were involved in FAK/AKT pathway. COL6A5 and COL9A2 are members of novel ECM proteins to form microfibrillar network. COL9A2 constitutes the largest component of ECM as collagen “skeleton”, whereas the expression, with more evidences, of COL6A5 is restricted to kidney, skin, skeletal muscle glomerulus, interfollicular stroma in ovary or testis [54, 55]. Both of the two molecules play crucial roles in many degenerative disorders or inflammatory diseases like osteoarthritis, dermatitis and so on [56, 57]. The abnormity of these ECM molecules in quality or quantity will trigger structural or functional disturbance of target organs. In ovaries, germ cells maturation and follicles development rely much on the re-organization and modification of ECM to provide major collagen framework for ovarian tissue and serve as transportation hub for nutrients, hormones and signaling molecules [58, 59]. As indicated in this study, the expression of COL6A5 and COL9A2 were down-regulated after CTX treatment, which signified the destructive effect of CTX on ovarian structure and function. Conversely, MenSCs graft could finally recover the expression of these two structural molecules and improved ovarian status. In this research, we also found FAK was activated at either RNA or protein levels. FAK is a significant mediator of ECM signaling in the regulation of cell survival and proliferation, many studies have revealed that ECM could result in different cellular behaviors via activating FAK [60]. Consistent with these findings, our results demonstrated activated FAK could later promote the phosphorylation changes in AKT. Then, the activation of AKT would probably inhibit its target molecules including NR4A1 and CDKN1A. NR4A1 was involved in cell survival or apoptosis, DNA repair, and tumorigenesis [61–63], and CDKN1A was involved in arresting cell cycle [64, 65]. After MenSCs transplantation, the phosphorylation modification of these molecules was dramatically reduced, thus leading to inhibition of apoptosis and cell cycle arrest as shown in Hoechst and TUNEL assay results. It might account for the homeostasis regulation in ovarian microenvironment to relieve ovaries from CTX damage after MenSCs transplantation. Taken together, MenSCs participated in the regulation of ovarian function in ECM-dependent FAK-AKT signaling pathway.

## Conclusions

The efficacy of MenSCs in the treatment of POI lends considerable therapeutic potential with great advantages of non-invasiveness, robust source and low immunogenicity, over other sources of MSCs. Additionally, our study focuses on the reparative effects of MenSCs on POI mouse models, yet it is still not known whether our results are applicable to further studies in other higher mammals or even human beings. It should be pointed out in particular that POI patients are usually amenorrheic clinically and it is hard for them to collect menstrual blood from normally spontaneous menstruation. In these patients, artificial menstrual cycles are often adopted to sustain normal function of their uteri and thus they regain menstruation. However, whether can MenSCs from normal woman or POI patients exhibit same effect is still unknown and it is in urgent need of more researches in POI treatment to compare the similarities and differences of MenSCs between normal donors and POI patients. Apart from this, it is necessary to explore the tumorigenicity of MenSCs transplantation in vivo with follow-up for long time to comprehensively assess the safety of this treatment. Certainly, MenSCs therapy has a great potential in the future.

## Electronic supplementary material

Fig. S1 Identification and Characterization of MenSCs. **A.** Representative picture of MenSCs morphology at passage two. Original magnification, ×40. **B.** Histograms of flow cytometry analysis for expression of surface markers on MenSCs (high expression of MSCs markers CD105, CD90, CD73 and CD44 but low expression of hematopoietic cells markers CD45, CD38 and CD34). C**.** Adipogenic induction differentiation of MenSCs was visualized by Oil Red O staining for lipid droplets. Original magnification, ×200. **D.** Chondrogenic differentiation of MenSCs was observed by Alcian Blue staining for mucopolysaccharide. Original magnification, ×200. **E.** Osteogenic differentiation of MenSCs was identified by Alizarin red staining for calcium deposition. Original magnification, ×200. **F.** Proliferation curves of MenSCs at P2 or P10 were compared by CCK-8 assay. G. Representative picture of CFU with more than 50 cells under microscope. Original magnification, ×200. **H.** CFU numbers of MenSCs at P2 or P10 were assessed, *n* = 6. Data were represented as mean ± SEM. (PNG 2011 kb)

High Resolution (TIF 13262 kb)

ESM 1 (DOC 28 kb)

Table S1 (DOC 48 kb)

## Abbreviations

*POI*: premature ovarian insufficiency
*MenSCs*: human menstrual blood-derived stromal cells
*MSCs*: mesenchymal stem cells
*CTX*: cyclophosphamide
*E2*: estradiol
*AMH*: anti-Mullerian hormone
*FSH*: follicle-stimulating hormone
*P2*: passage 2
*P10*: passage 10
*PBS*: phosphate buffered saline
*CCK-8*: cell-counting kit 8
*OD*: optimal density
*CFU*: colony-forming units
*P*: proestrus
*E*: estrus
*Me*: metaestrus
*Di*: diestrus
*H&E*: hematoxylin and eosin
*ELISA*: enzyme linked immunosorbent assay
*TUNEL*: terminal deoxynucleotidyl transferase dUTP nick end labeling
*q-RT PCR*: quantitative real-time PCR
*IHC*: immunohistochemistry
*DEGs*: differentially expressed genes
*RPKM*: Reads per Kilobase per Million Reads
*CPM*: counts per million reads
*GAPDH*: glyceraldehyde-3-phosphate dehydrogenase
*DDX4*: DEAD box polypeptide 4
*VEGFA*: vascular endothelial growth factor A
*COL6A5*: collagen type VI alpha 5 chain
*COL9A2*: collagen type IX alpha 2 chain
*FAK*: focal adhesion kinase
*AKT*: serine-threonine protein kinase
*NR4A1*: nuclear receptor subfamily 4 group A member 1
*CDKN1A*: cyclin dependent kinase inhibitor 1A
*ECM*: extracellular matrix
