# Supplementary figures and images for: Human Menstrual Blood-Derived Stromal Cells Promote Recovery of Premature Ovarian Insufficiency Via Regulating the ECM-Dependent FAK/AKT Signaling
Source: Stem Cell Rev. 2018 Dec 17;15(2):241–55. doi: 10.1007/s12015-018-9867-0 (PMC6441404; doi:10.1007/s12015-018-9867-0)

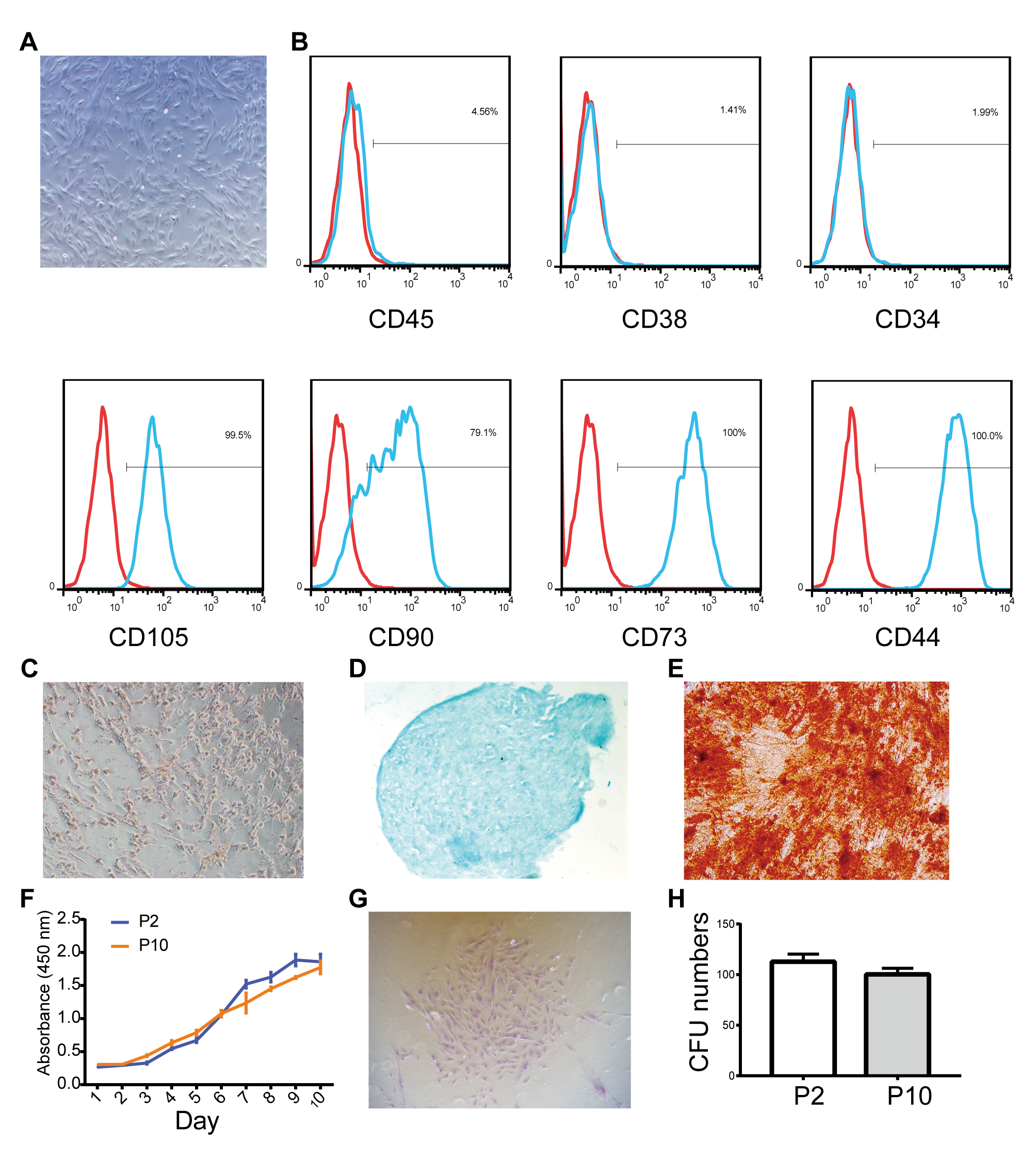

Supplement: Supplementary file 1 — Identification and Characterization of MenSCs. A. Representative picture of MenSCs morphology at passage two. Original magnification, ×40. B. Histograms of flow cytometry analysis for expression of surface markers on MenSCs (high expression of MSCs markers CD105, CD90, CD73 and CD44 but low expression of hematopoietic cells markers CD45, CD38 and CD34). C. Adipogenic induction differentiation of MenSCs was visualized by Oil Red O staining for lipid droplets. Original magnification, ×200. D. Chondrogenic differentiation of MenSCs was observed by Alcian Blue staining for mucopolysaccharide. Original magnification, ×200. E. Osteogenic differentiation of MenSCs was identified by Alizarin red staining for calcium deposition. Original magnification, ×200. F. Proliferation curves of MenSCs at P2 or P10 were compared by CCK-8 assay. G. Representative picture of CFU with more than 50 cells under microscope. Original magnification, ×200. H. CFU numbers of MenSCs at P2 or P10 were assessed, n = 6. Data were represented as mean ± SEM. (PNG 2011 kb) [file 12015_2018_9867_Fig6_ESM.png]

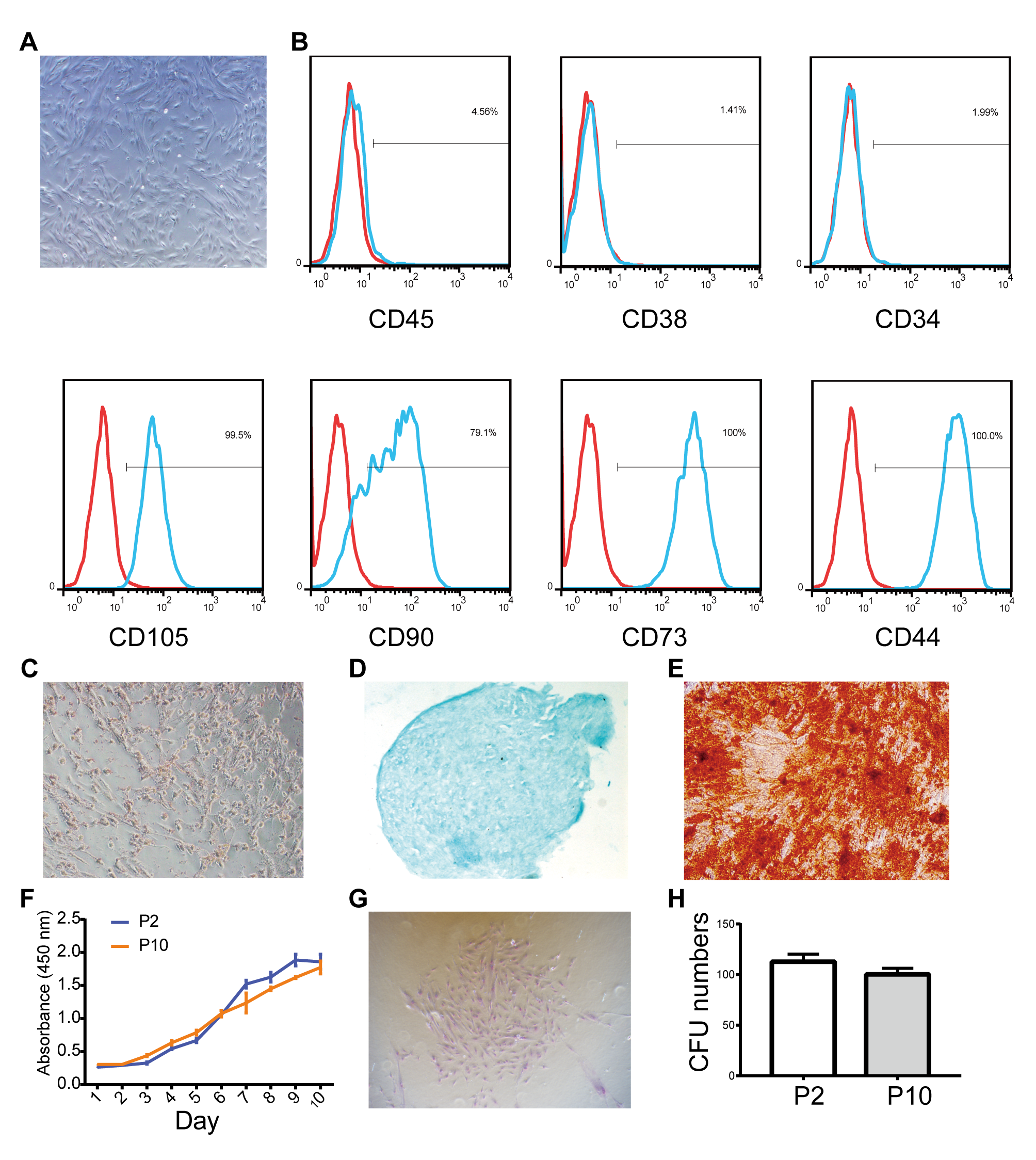

Supplement: Supplementary file 2 — High Resolution (TIF 13262 kb) [file 12015_2018_9867_MOESM1_ESM.tif]
