# Supplementary material for: Human Menstrual Blood-Derived Stromal Cells Promote Recovery of Premature Ovarian Insufficiency Via Regulating the ECM-Dependent FAK/AKT Signaling
Source: Stem Cell Rev. 2018 Dec 17;15(2):241–55. doi: 10.1007/s12015-018-9867-0 (PMC6441404; doi:10.1007/s12015-018-9867-0)
Supplement: Supplementary file 3 — (DOC 28 kb) [file 12015_2018_9867_MOESM2_ESM.doc]

**Western blotting analysis**

Ovarian tissues were homogenized with RIPA lysis buffer (Beyotime, Beijing, China) containing 1 mM PMSF and a mixture of phosphatase inhibitors. The supernatant was collected after centrifugation, and protein concentration was assessed by BCA method. Quantitative protein was subjected to SDS-PAGE in polyacrylamide gel after protein degeneration for 8 min at 95 °C, which was subsequently transferred onto polyvinylidene difluoride membranes (Millipore, MA, USA) for 90 min at 200 mA. Later, the membranes were blocked in 5% skim milk in Tris-HCl buffer solution containing 0.1% Tween-20 for one hour at room temperature and were separately immunoblotted with primary antibodies at 4 °C overnight against AMH, DDX4, VEGFA COL6A5, COL9A2, FAK, Phospho-FAK (Tyr861), AKT, Phospho-AKT (Thr308), NR4A1, Phospho-NR4A1 (Ser351), CDKN1A, Phospho-CDKN1A (Ser146) and GAPDH. After three rinses, the membranes were incubated with HRP-conjugated anti-mouse or anti-rabbit IgG secondary antibody for one hour at room temperature. The blots were finally visualized using an enhanced electrochemiluminescence (ECL) kit (Thermo Fisher, CA, USA), and the gray-scale analysis was determined via Image J software (National Institutes of Health, Bethesda, MD, USA) with GAPDH as reference protein for normalization.
