# Supplementary material for: Human Menstrual Blood-Derived Stromal Cells Promote Recovery of Premature Ovarian Insufficiency Via Regulating the ECM-Dependent FAK/AKT Signaling
Source: Stem Cell Rev. 2018 Dec 17;15(2):241–55. doi: 10.1007/s12015-018-9867-0 (PMC6441404; doi:10.1007/s12015-018-9867-0)
Supplement: Supplementary file 4 — (DOC 48 kb) [file 12015_2018_9867_MOESM3_ESM.doc]

**Table. S1 Product information of antibodies**

| **Serial number** | **Kits and reagents** | **Manufacturers** | **Catalogue number** | **Place of origin** |
| --- | --- | --- | --- | --- |
| 1 | CD34 antibody | BD Biosciences | 555821 | Franklin Lakes, NJ, USA |
| 2 | CD38 antibody | BD Biosciences | 555460 | Franklin Lakes, NJ, USA |
| 3 | CD44 antibody | BD Biosciences | 555478 | Franklin Lakes, NJ, USA |
| 4 | CD45 antibody | BD Biosciences | 555482 | Franklin Lakes, NJ, USA |
| 5 | CD73 antibody | BD Biosciences | 550257 | Franklin Lakes, NJ, USA |
| 6 | CD90 antibody | BD Biosciences | 555596 | Franklin Lakes, NJ, USA |
| 7 | CD105 antibody | BD Biosciences | 560839 | Franklin Lakes, NJ, USA |
| 8 | AMH antibody | Santa Cruz | sc-166752 | CA, USA |
| 9 | DDX4 antibody | Abcam | ab13840 | MA, USA |
| 10 | VEGFA antibody | Novus | NB100-664 | CA, USA |
| 11 | COL6A5 antibody | Invitrogen | PA5-70781 | CA, USA |
| 12 | COL9A2 antibody | Santa Cruz | sc-398130 | CA, USA |
| 13 | FAK antibody | Wanleibio | WL01748 | Beijing, China |
| 14 | Phospho-FAK (Tyr861) antibody | Absin | abs131024 | Shanghai, China |
| 15 | AKT antibody | CST | 4691T | MA, USA |
| 16 | Phospho-AKT (Thr308) antibody | CST | 13038T | MA, USA |
| 17 | NR4A1 antibody | Santa Cruz | sc-365113 | CA, USA |
| 18 | Phospho-NR4A1 (Ser351) antibody | Absin | abs106402 | Shanghai, China |
| 19 | CDKN1A antibody | Santa Cruz | sc-6246 | CA, USA |
| 20 | Phospho-CDKN1A (Ser146) antibody | Santa Cruz | sc-377515 | CA, USA |
| 21 | GAPDH antibody | CST | 97166S | MA, USA |
